# Anxiety, depression and health-related quality of life in patients with thoracic aortic disease: a longitudinal study in a cardiothoracic outpatient clinic

**DOI:** 10.3389/fcvm.2026.1715132

**Published:** 2026-06-29

**Authors:** Ismail Dalyanoglu, Anna Maria Markser, Johannes Nienhaus, Esma Yilmaz, Mohammed Morjan, Amin Thwairan, Ulrike Dinger, Artur Lichtenberg, Hannan Dalyanoglu

**Affiliations:** 1 Department for Vascular and Endovascular Surgery, Sankt Marien-Hospital Buer GmbH, Gelsenkirchen, Germany; 2 Clinical Institute for Psychosomatic Medicine and Psychotherapy, University Hospital Duesseldorf, Duesseldorf, Germany; 3 Department of Anesthesiology, University Hospital Duesseldorf, Duesseldorf, Germany; 4 Department of Cardiac Surgery, University Hospital Duesseldorf, Duesseldorf, Germany

**Keywords:** EQ-5D-5L, HRQoL, mixed-effects models, PHQ-4, psychological distress, thoracic aortic disease

## Abstract

**Background:**

Thoracic aortic diseases (TAD) require complex surgical and longitudinal follow-up care. Evidence on health-related quality of life (HRQoL) and psychological distress in real-world TAD outpatient cohorts remains limited.

**Objective:**

To describe longitudinal trajectories of HRQoL and psychological distress in patients with TAD and to examine associations with key demographic and clinical variables.

**Methods:**

We retrospectively analyzed 111 patients (256 visits, 2023–2025). Primary outcomes were EQ-5D-5L index values, EQ-VAS scores, and PHQ-4 total scores. Linear mixed-effects models (random intercepts for patient ID) and logistic regression (PHQ-4 ≥6) were used to account for repeated measures. Primary predictors included sex, surgical status, and time since surgery; age and visit number were examined exploratorily.

**Results:**

Median EQ-5D-5L was 0.71 (IQR 0.55–0.88), EQ-VAS 70 (60–80), and PHQ-4 3 (1–5). Time since surgery predicted HRQoL improvement (*β* > 0, *p* < 0.001). Preoperative patients showed lower HRQoL (*β* < 0, *p* < 0.01) and twice the odds of clinically relevant distress (OR 2.0, 95% CI 1.2–3.3). Female sex showed nonsignificant trends toward lower HRQoL and higher distress.

**Conclusions:**

Preoperative TAD patients reported lower HRQoL and higher distress, while postoperative recovery was associated with gradual improvement. Findings reflect real-world patterns in a mixed pre-/postoperative outpatient cohort and support integrating brief psychosocial screening into routine TAD follow-up.

## Introduction

Thoracic aortic diseases (TAD), including aneurysms and dissections, are life-threatening conditions that require complex surgical and long-term outpatient management beyond mere procedural success (1, 2). In addition to considerable perioperative risk, patients often experience persistent uncertainty, lifelong imaging surveillance, and concern about potential reinterventions, all of which may contribute to psychological burden. Previous work has described psychosocial sequelae after aortic interventions (3–6), yet longitudinal data specifically focusing on patient-reported outcomes in TAD remain scarce. Findings from abdominal aortic aneurysm (AAA) cohorts show peaks in preoperative anxiety followed by postoperative improvement, although these results cannot be fully transferred to TAD because of disease-specific characteristics, including hereditary syndromes, younger age at diagnosis in selected subgroups, and the complexity of cardiothoracic procedures (6–10). Existing studies in TAD populations indicate reduced health-related quality of life (HRQoL) compared with normative populations (11, 12), consistent with findings in other cardiac surgical groups such as patients undergoing CABG procedures (13–15), where longitudinal assessments demonstrate heterogeneous recovery trajectories (16, 17). Recent longitudinal analyses using EQ-5D-5L in aortic cohorts further underline the relevance of patient-reported outcomes in this population (4, 18). Despite these insights, psychosocial assessment has not been systematically integrated into routine TAD follow-up care, and real-world longitudinal data from specialized aortic outpatient clinics remain limited. This study therefore aimed to characterize longitudinal trajectories of HRQoL and psychological distress in patients with thoracic aortic disease treated in a specialized cardiothoracic outpatient clinic. We hypothesized that postoperative patients would report improved HRQoL and lower psychological distress compared with preoperative patients. In addition, we conducted exploratory analyses to identify potential subgroups with divergent psychosocial trajectories. This observational real-world study was designed to generate descriptive and hypothesis-oriented insights while evaluating the feasibility of integrating brief psychosocial screening tools into routine aortic outpatient care.

## Materials and methods

### Study design and ethical approval

This retrospective longitudinal study included all patients presenting to the aortic outpatient clinic of the University Hospital Düsseldorf Cardiac Surgery Center between 2023 and 2025. The dataset comprised 111 patients with a total of 256 clinical visits, including longitudinal observations from 67 patients with multiple assessments as well as cross-sectional data from single-visit patients. All available data were analyzed without imputation to maximize information while maintaining methodological rigor.

Data were fully de-identified and pseudonymized through unique study identifiers, enabling longitudinal linkage without providing investigators access to directly identifiable information. The study protocol (2023–2566) was approved by the Ethics Committee of Heinrich Heine University Düsseldorf on December 6, 2023, and conducted in accordance with the Declaration of Helsinki, the General Data Protection Regulation (GDPR), and STROBE reporting guidelines. Individual informed consent was waived for this analysis of pseudonymized routine clinical data.

This dataset reflects a real-world outpatient cohort with heterogeneous pre- and postoperative trajectories rather than a predefined comparative trial. Pre- and postoperative classifications refer to the clinical status at the time of presentation to the aortic outpatient clinic rather than a strictly longitudinal within-patient pre- to post-surgical follow-up. Due to real-world referral patterns, including second opinions at other institutions and irregular follow-up intervals, the proportion of pre- and postoperative visits is naturally unbalanced and does not represent a paired transition within the same individuals. Accordingly, analyses were planned to adjust for key clinical variables (e.g., surgical status, time since surgery) and to generate descriptive, hypothesis-oriented insights.

### Population and eligibility criteria

**Figure 1:**
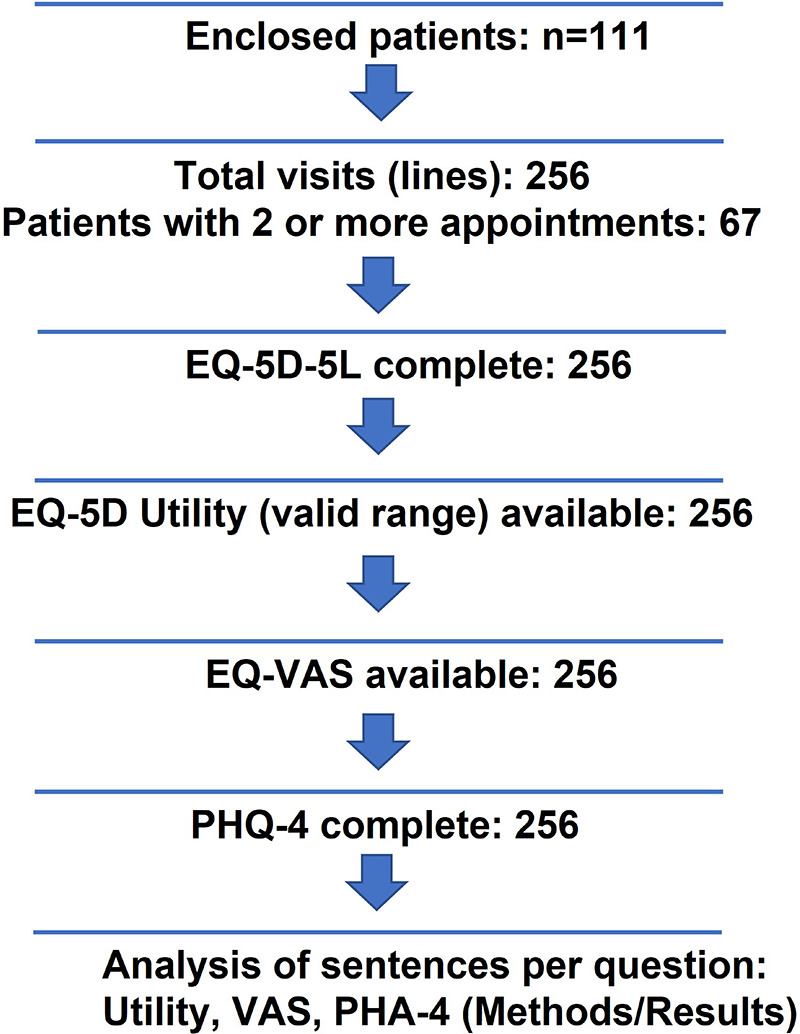
STROBE-compliant flowchart of selection and exclusions.

All consecutive patients aged 18 years or older presenting to the aortic outpatient clinic during the study period were eligible. Inclusion required completion of at least one full assessment consisting of the EQ-5D-5L (including EQ-VAS), the PHQ-4, and a valid pseudonymized identifier. Exclusion criteria were incomplete core demographic data (age, sex, or visit date) or documented withdrawal of consent for secondary data use. The patient selection process and exclusion reasons are summarized in a STROBE-compliant flowchart (Figure 1).

Preoperative status was defined as any clinic visit before planned or acute thoracic aortic surgery. Postoperative status included all follow-up visits after completed thoracic aortic procedures. Sex was coded dichotomously (female = 1; male = 0), and surgical status as preoperative = 1 vs. postoperative = 0. Because patients with acute aortic dissection rarely undergo preoperative assessment, most were included postoperatively; thus, inferential pre- vs. postoperative comparisons were not planned for that subgroup.

### Instruments

HRQoL was assessed using the EQ-5D-5L instrument, including the EQ-VAS component (19). The EQ-5D-5L evaluates mobility, self-care, usual activities, pain/discomfort, and anxiety/depression across five severity levels, generating an index score using the German valuation set (range −0.661 to 1). The EQ-VAS provides a global self-rated health measure (0 = worst imaginable health, 100 = best).

Psychological distress was assessed using the PHQ-4, a validated four-item screener for anxiety and depressive symptoms (1, 9, 10). PHQ-4 scores (0–12) were categorized into 0–2, 3–5, 6–8, and 9–12; scores ≥6 indicated clinically relevant distress for logistic regression.

The primary outcomes were the EQ-5D-5L index, EQ-VAS, and the PHQ-4 total score. These tools were chosen for their brevity, validity in surgical cohorts, and suitability for repeated assessment in a busy outpatient setting.

### Predictor variables

Key predictors included age (continuous, years), biological sex, surgical status, time since surgery (continuous days from aortic procedure to assessment; preoperative visits coded as 0 days), and sequential visit number (1 = first visit, maximum 8).

### Data cleaning and missing data

Data were systematically screened for plausibility and completeness. Missing data were limited (EQ-5D-5L 9%; PHQ-4 and EQ-VAS complete) and showed no systematic pattern, supporting a missing-at-random assumption. Complete-case analysis was performed. Further details on data cleaning procedures are provided in the Supplementary Appendix.

### Statistical analysis

Due to non-normal outcome distributions, non-parametric correlations (Spearman) and mixed-effects models with random intercepts for patient ID were applied to account for repeated measures. The binary outcome (PHQ-4 ≥6) was analyzed using logistic regression with cluster-robust standard errors.

Model specification followed a predefined analysis plan and was restricted to main effects to avoid overfitting. Model assumptions were assessed and indicated adequate model fit (see Supplementary Appendix for detailed diagnostics).

### Exploratory pattern analysis

An exploratory unsupervised analysis was conducted among patients with ≥2 visits to identify longitudinal patterns in HRQoL and psychological distress. Dimensionality reduction and clustering techniques were applied, yielding three descriptive trajectory patterns. Detailed information on the analytical procedure is provided in the Supplementary Appendix.

All analyses utilized R version 4.3 with packages lme4, cluster, and stats. A two-sided significance level of 0.05 was applied. Model development strictly followed the pre-specified plan; no *post-hoc* analyses or data-driven model modifications were performed.

## Results

### Demographic characteristics

**Figure 2:**
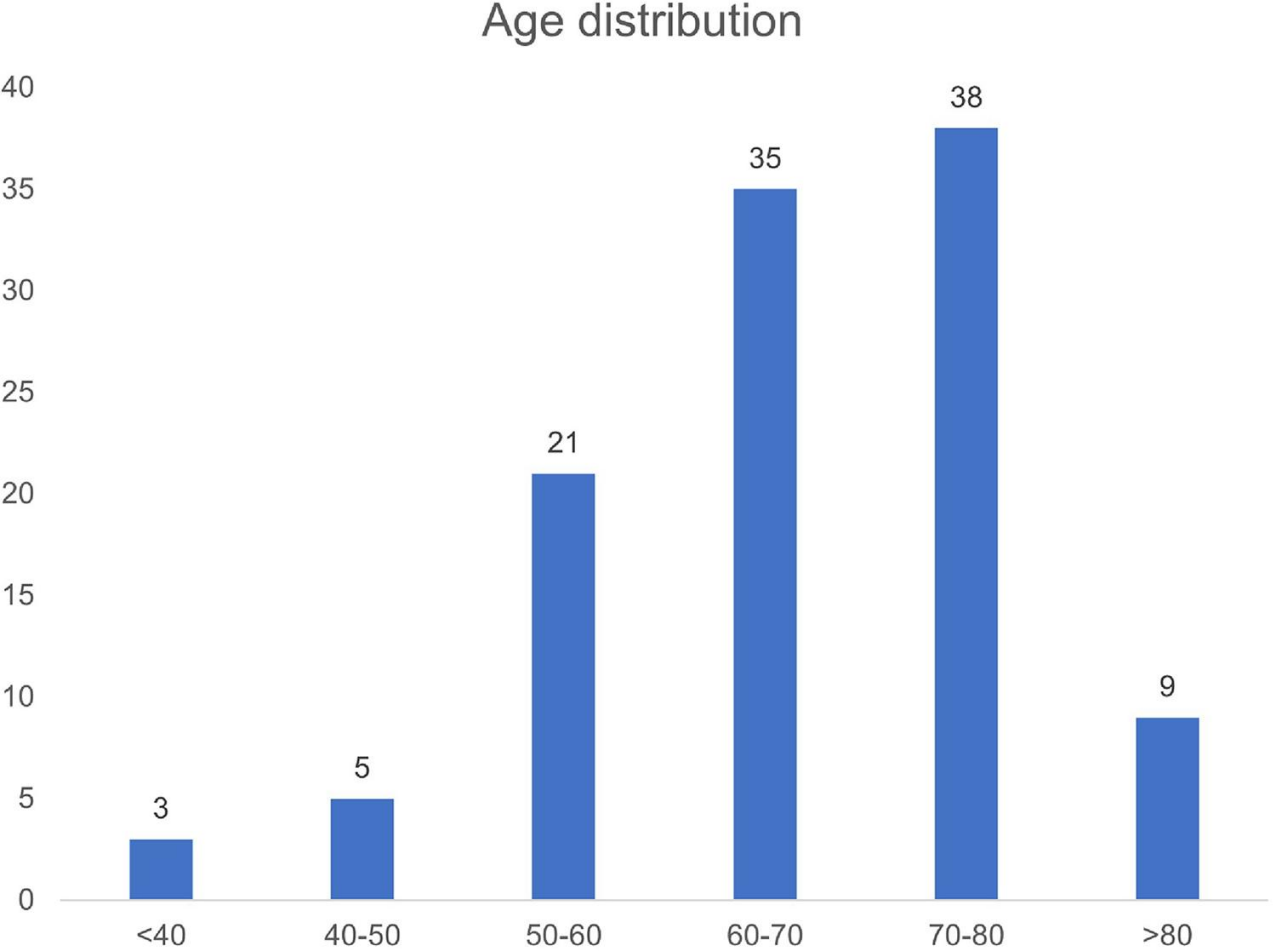
Age distribution (*n* = 111) showing range 32–85 years with patient counts per age group.

**Figure 3:**
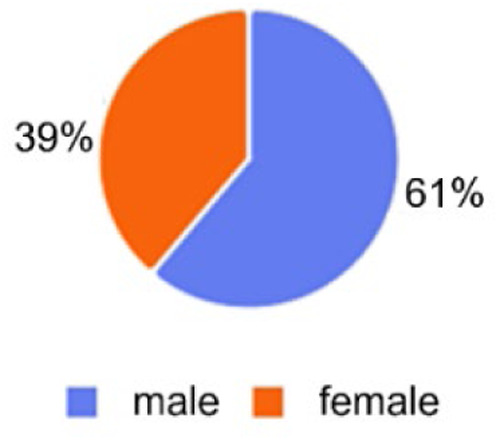
Sex distribution (68 male, 43 female).

**Figure 4:**
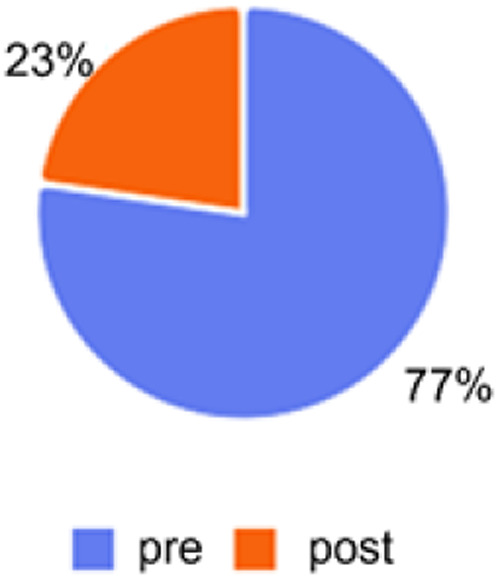
Summarizes surgical status at initia/first presentation (pre = preoperative, post = postoperative, excluded: missing core data, excluded: withdrawal of permission.

The cohort included 111 patients (68 male, 43 female; mean age 64.8 ± 11.1 years, range 32–85) who contributed 256 outpatient visits between 2023 and 2025. Of these, 67 patients had longitudinal data with at least two visits. At first presentation, 77% of patients were preoperative. Baseline demographic and clinical characteristics by surgical status are summarized in Table 1, and the patient selection process is shown in the STROBE-compliant flowchart (Figures 1–4).

**Table 1:** Demographic data at initial presentation.

Variable	Sex	Number(n)	Mean Age (year)	Min (years)	Max (years)
Patients		111			
	Male	68 (61%)			
	Female	43 (39%)			
Overall Mean Age			64.8	32	85
Preoperative		85 (77%)			
	Male	35 (41%)	63.1	38	82
	Female	50 (59%)	67.5	32	84
Postoperative		26 (23%)			
	Male	14 (54%)	62.2	42	70
	Female	12 (46%)	71.5	50	85

### Baseline characteristics by surgical Status

Descriptive comparisons between operated and non-operated patients (Table 2) showed no significant differences in age (65.8 vs. 63.8 years; *p* = 0.482) or sex distribution. HRQoL, however, was higher in the operated group (EQ-5D-5L 0.51 vs. 0.14; *p* = 0.008). Psychological distress (PHQ-4: 1.92 vs. 2.55; *p* = 0.414) did not differ significantly. Pain intensity (VAS) was higher in non-operated patients (53.8 vs. 63.5; *p* = 0.038). These differences likely reflect real-world clinical selection mechanisms and should be interpreted descriptively (Table 2). Overall, while the groups were demographically comparable, they differed in baseline HRQoL and pain levels. These disparities should be considered when interpreting subsequent outcome analyses, as they may reflect underlying selection mechanisms or residual confounding. These descriptive differences illustrate the selection mechanisms inherent in real-world surgical decision-making and highlight that patients eligible for surgery differ from those managed conservatively. However, due to sample size limitations and the observational design, we did not perform fully adjusted comparative outcome models between these subgroups, and all subgroup contrasts should be interpreted descriptively.

**Table 2:** Baseline characteristics stratified by whether patients underwent surgery.

Variable	Operated (*n* = 25)	Not-operated (*n* = 86)	*p*-Value
Age	65.8	63.8	0.482
Gender			
M	13 (52.0%)	55 (64.0%)	
W	12 (48.0%)	31 (36.0%)	
Baseline HRQoL	0.51	0.14	0.008
Baseline PHQ-4	1.92	2.55	0.414
VAS	53.8	63.5	0.038

### Baseline characteristics: dissection vs. Aneurysm

Patients presenting with dissection (*n* = 17) and aneurysm (*n* = 94) showed no significant differences in age (65.6 vs. 64.0 years; *p* = 0.631) or sex distribution. HRQoL at baseline was higher in dissection patients (0.51 vs. 0.14; *p* = 0.005), while pain levels were higher in aneurysm patients (53.8 vs. 63.5; *p* = 0.039). PHQ-4 scores did not differ significantly (1.92 vs. 2.55; *p* = 0.305). Given that most dissection patients were assessed postoperatively, these subgroup differences are descriptive and not suited for inferential comparison (Table 3). Overall, while demographic characteristics were largely comparable, the two groups differed significantly in baseline HRQoL and pain intensity. These differences should be taken into account when interpreting subsequent outcome analyses, as they may indicate underlying differences in disease presentation, symptom burden, and patient trajectories. The dissection subgroup thus represents a clinically distinct group that is predominantly captured after emergency repair. While this supports the clinical intuition that dissected and aneurysmal patients differ in several respects, the available data do not permit robust inferential pre- vs. postoperative comparisons within the dissection subgroup. These contrasts illustrate real-world selection but should be interpreted as descriptive rather than inferential.

**Table 3:** Baseline characteristics of patients with aortic dissection vs. aneurysm.

Variable	Dissection (*n* = 17)	Aneurysm (*n* = 94)	*p*-Value
Age	65.6	64.0	0.631
Gender			
M	12 (70.6%)	56 (59.6%)	
W	5 (29.4%)	38 (40.4%)	
Baseline HRQoL	0.51	0.14	0.005
Baseline PHQ-4	1.92	2.55	0.305
VAS	53.8	63.5	0.039

#### Outcome distributions

**Figure 5:**
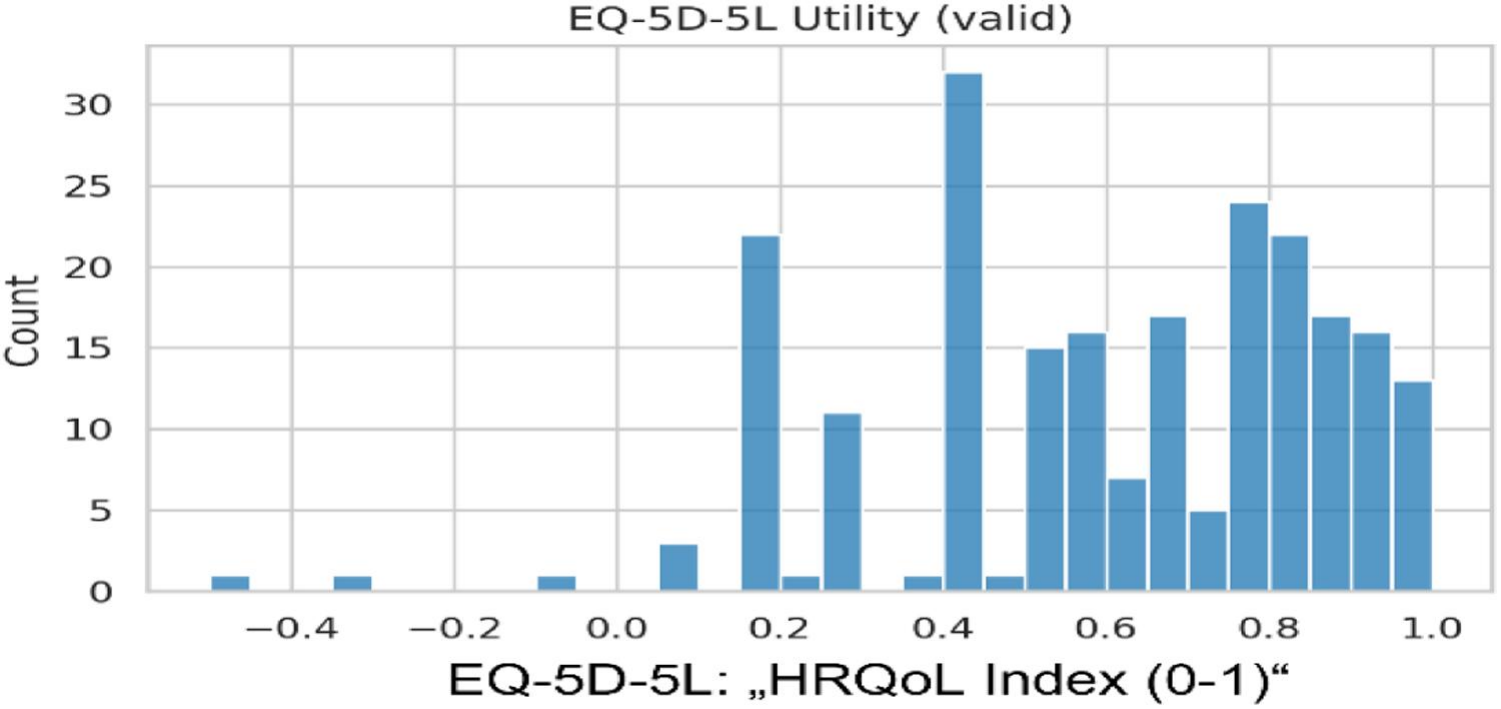
Distribution of EQ-5D-5L index values using the German tariff. The distribution is left-skewed with peak values around 0.9 and tail extending to −0.5, indicating most patients reported moderate to good health states (0.55-0.9).

**Figure 6:**
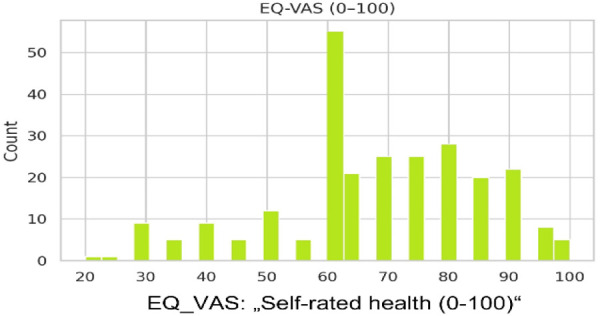
Histogram of EQ-VAS (0–100 scale) scores. Distribution of subjective health. EQ-5D-5L index (0–1, higher = better HRQoL); EQ-VAS (0–100, higher = better). EQ-5D-5L: left-skewed (peak ∼0.9, tail to −0.5).

**Figure 7:**
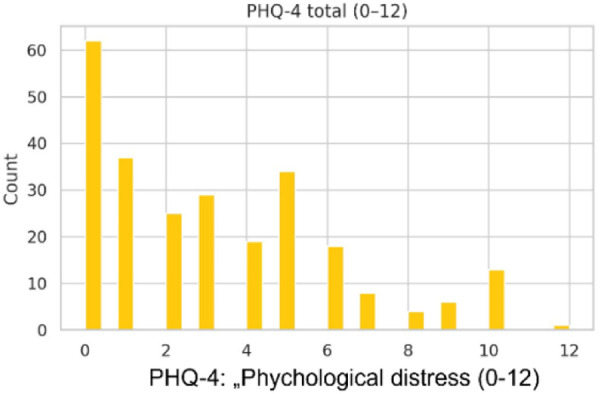
Distribution of PHQ-4 total scores (0–12). The histogram exhibits a right-skewed pattern with dominant peak at 0–1, steadily decreasing frequencies toward higher scores, and relatively few observations in the 6–12 range, confirming most scores fall within normal-to-mild distress while a minority reaches moderate-to-severe levels.

**Figure 8:**
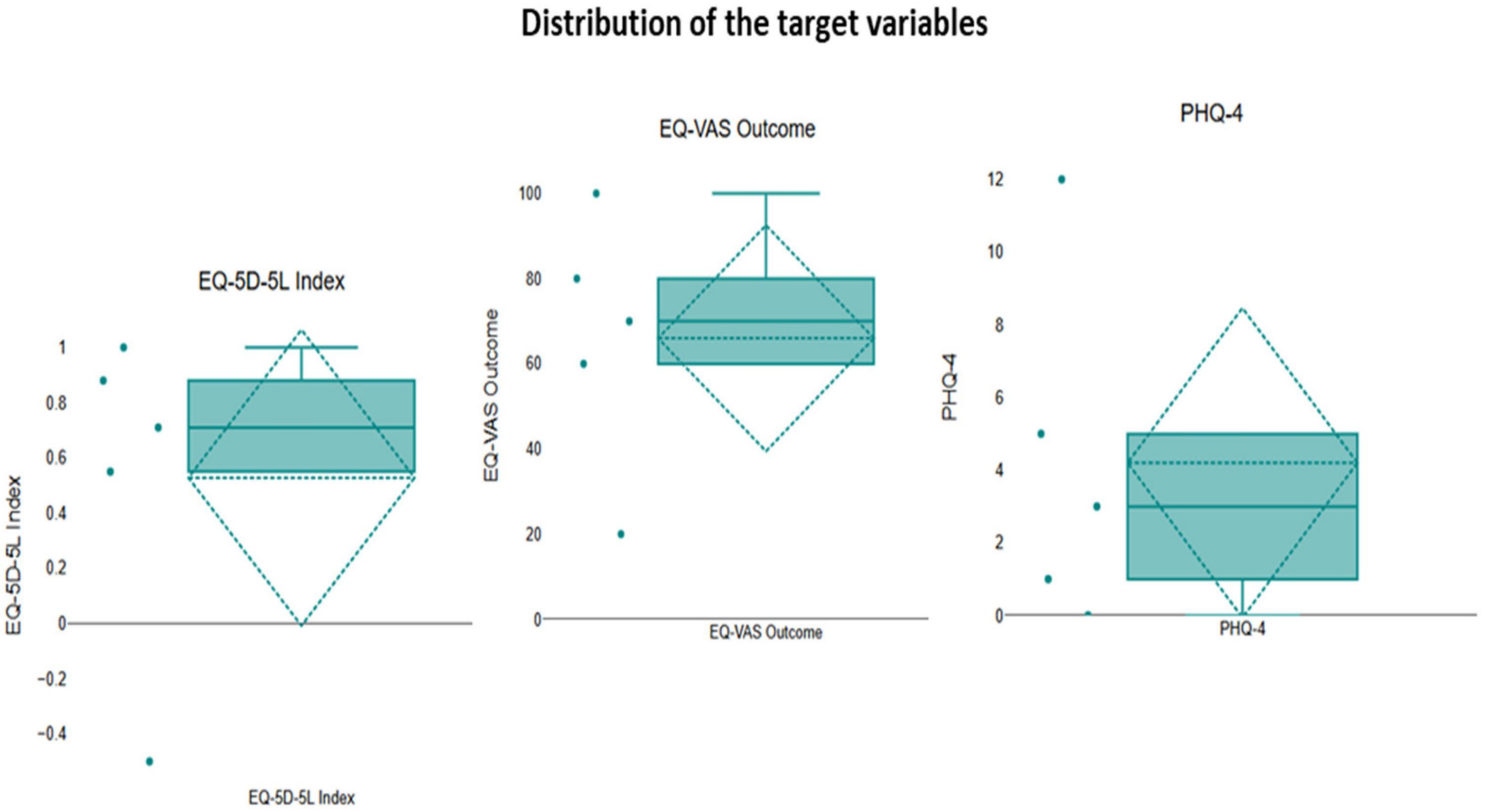
Boxplots of primary outcomes across 256 visits (EQ-5D-5L index left-skewed median ∼0.7; EQ-VAS symmetric median 70 IQR 60–80; PHQ-4 right-skewed median ∼3 tail to 12). Substantial inter-individual variability evident, particularly in PHQ-4 distress scores.

Outcome variables and descriptive statistics (Table 4, Figures 5–8) show moderate HRQoL (EQ-5D-5L median 0.71 [IQR 0.55–0.88]; EQ-VAS 70 [60–80]) and low-to-moderate distress (PHQ-4 3.0 [1–5]) across 256 visits. EQ-5D-5L index ranged from severely impaired (≤0.5) to near-optimal (≥0.9) health states, EQ-VAS clustered between 60 and 85, and PHQ-4 was right-skewed (mode = 0, tail to 12) with many low scores and a long tail toward higher values (normal-to-mild range (0–5), moderate-severe range (≥6).

**Table 4:** Outcomes—descriptive values (*n* = number; mean = mean; SD, standard deviation; IQR_low, interquartile range Low; IQR_high, interquartile range high).

Outcome	*n*	Median	IQR (Q1—Q3)	Range (min—max)
EQ-VAS	256	70	60.0–80.0	20–100
PHQ-4	256	3.0	1.0–5.0	0–12
EQ-5D Utility (German tariff valid)	256	0.71	0.55–0.88	−0.661–1.0

Therefore, available-case analysis was used without imputation. Descriptive comparisons of baseline HRQoL and distress by sex, age group, and surgical status showed expected trends (e.g., younger and female patients had lower HRQoL and slightly higher distress), though these comparisons are not the primary focus and are detailed in Supplementary Analyses.

#### Descriptive and longitudinal statistics

Table 4 and Figures 5–8 summarize outcome distributions. EQ-5D-5L showed left-skewed distribution (peak −0.9, tail to −0.5), EQ-VAS clustered 60–85, and PHQ-4 exhibited right-skew (mode 0, tail to 12), with overall 20% clinically relevant distress (PHQ-4 ≥6).

EQ-VAS (0–100 scale) scores range from −20 to 100, peaking around 60 with additional peaks at 70–85; values <40 are rare (Figure 6).

PHQ-4 distribution was right-skewed (mode = 0, tail to 12) clinically relevant distress (PHQ-4 ≥6) occurred in 18.1% of preoperative visits (27/149) and 21.5% of postoperative visits (23/107), suggesting that a subset of patients reports clinically relevant distress at one or more visits (Figure 7).

Histograms (Figures 5–7) demonstrate that EQ-5D-5L left-skewed (peak −0.9, tail to −0.5), with most values near the upper range and a tail toward lower values. EQ-VAS (0–100 scale) values show a peak around 60–85, with few values falling below 40. PHQ-4 scores most frequently fall between 0 and 3, while only a minority reaches the upper range of the scale. These patterns show that, despite overall moderate-to-good health status, a subset of patients reports notable psychosocial burden or limited HRQoL.

Figure 8 demonstrates wide dispersion of EQ-5D-5L values, more stable EQ-VAS (0–100) scores, and substantial interindividual variability in PHQ-4. Shapiro–Wilk tests confirmed non-normality (EQ-5D *p* < 0.001, PHQ-4 *p* < 0.001, EQ-VAS *p* = 0.02). Thus medians/IQRs reported throughout; means/SD omitted for skewed outcomes.

#### Correlation analyses

Bivariate Spearman correlations revealed strong positive association between EQ-5D index and EQ-VAS (*ρ* = 0.75, *p* < 0.001) and modest negative correlations between PHQ-4 and both HRQoL measures. EQ-5D-5L index and PHQ-4 total score showed a modest negative correlation (*ρ* = −0.20, *p* < 0.001).

#### Longitudinal trajectories

**Figure 9:**
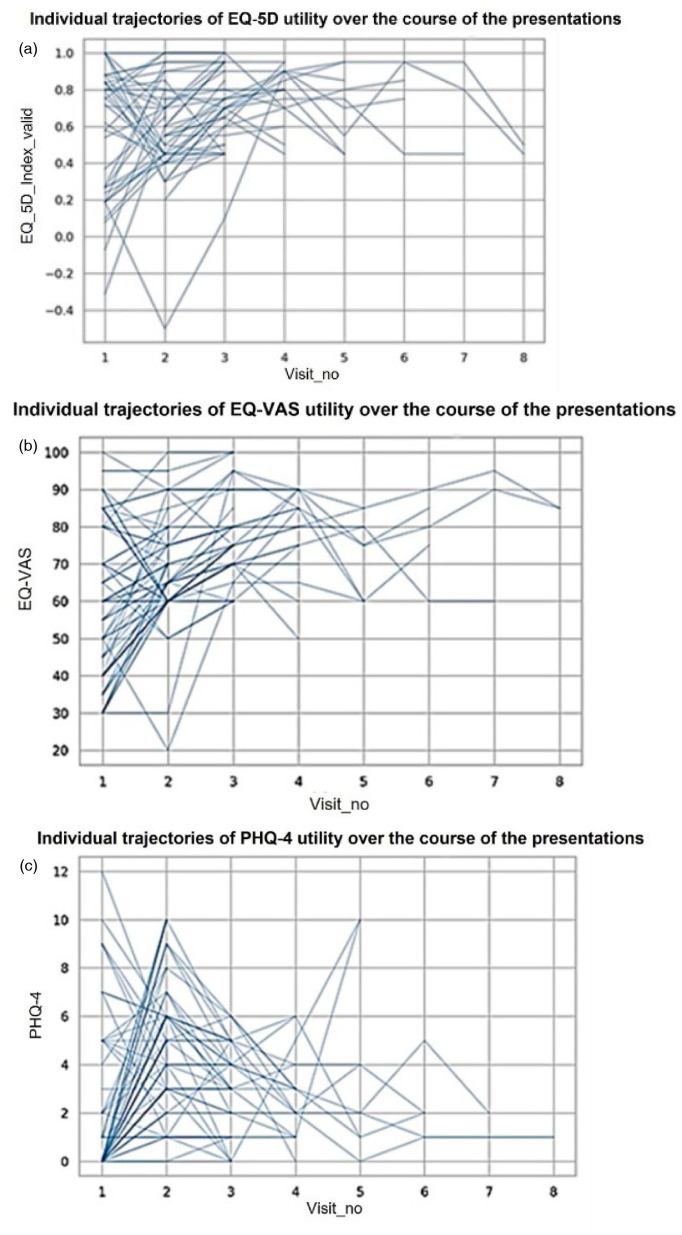
**(a)** individual trajectories of EQ-5D-5L index values across outpatient visits among patients with at least two assessments. Each line represents one patient. Most trajectories show stable or improving HRQoL over successive visits, with a minority exhibiting persistently low or declining scores, illustrating marked inter-individual heterogeneity. **(b)** Spaghetti plot/line plot with Individual trajectories of EQ-VAS scores (0–100) across outpatient visits among patients with at least two assessments. Each line represents one patient and illustrates substantial inter-individual variability, with most trajectories showing stable or improving self-rated health over time and a minority remaining in lower score ranges. **(c)** Spaghetti plot/line plot with Individual trajectories of PHQ-4 total scores (0–12) across outpatient visits among patients with at least two assessments. Each line represents one patient, revealing marked inter-individual heterogeneity: most trajectories show early peaks followed by decline over successive visits, while a minority exhibits persistent high distress or fluctuating patterns.

**Figure 10:**
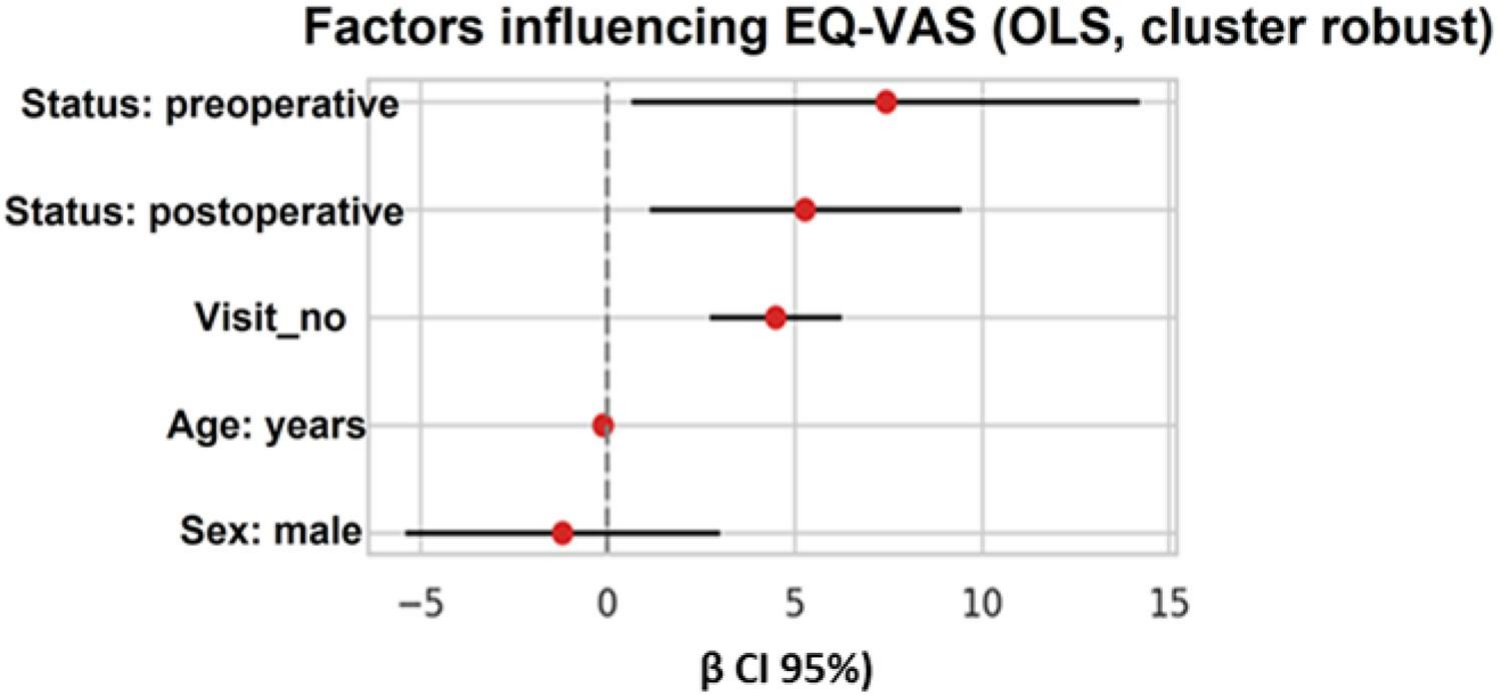
Forest plot of linear mixed-effects model *β*-coefficients (95% CI) for EQ-VAS (0–100). Model: EQ-VAS∼Time_since_surgery_days + Sex (0 = male ref, 1 = female) + Op_status (0 = postop ref, 1 = preop)+(1|Patient_ID). Positive *β* indicates improved subjective health; negative *β* indicates deterioration. Time since surgery and postoperative status are related to higher EQ-VAS scores, suggesting recovery over time, whereas female sex and preoperative status show trends toward lower perceived health. Clinically, this underscores the importance of postoperative psychosocial follow-up.

**Figure 11:**
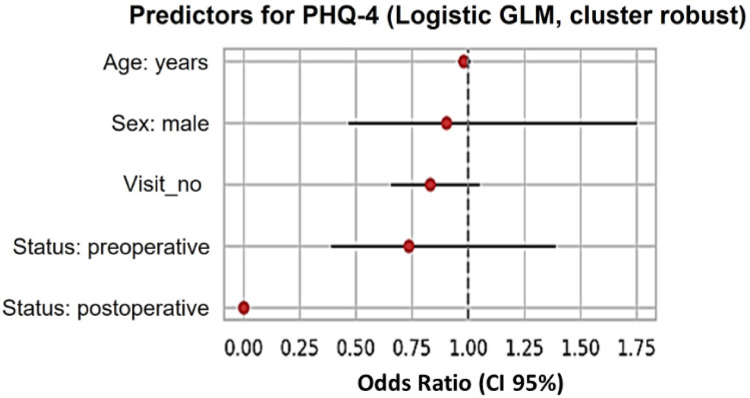
Forest plot of logistic regression odds ratios (95% CI) for PHQ-4 ≥6. Model predictors: age, sex (male vs. female reference), visit number, surgical status (pre- vs. postoperative reference). OR >1 indicates increased odds of clinically relevant distress; OR <1indicates reduced odds. Preoperative status (vs. postoperative) shows OR≈2.0 with CI excluding 1. Male sex shows protective trend (OR <1). Cluster-robust standard errors account for within-patient correlation.

**Figure 12:**
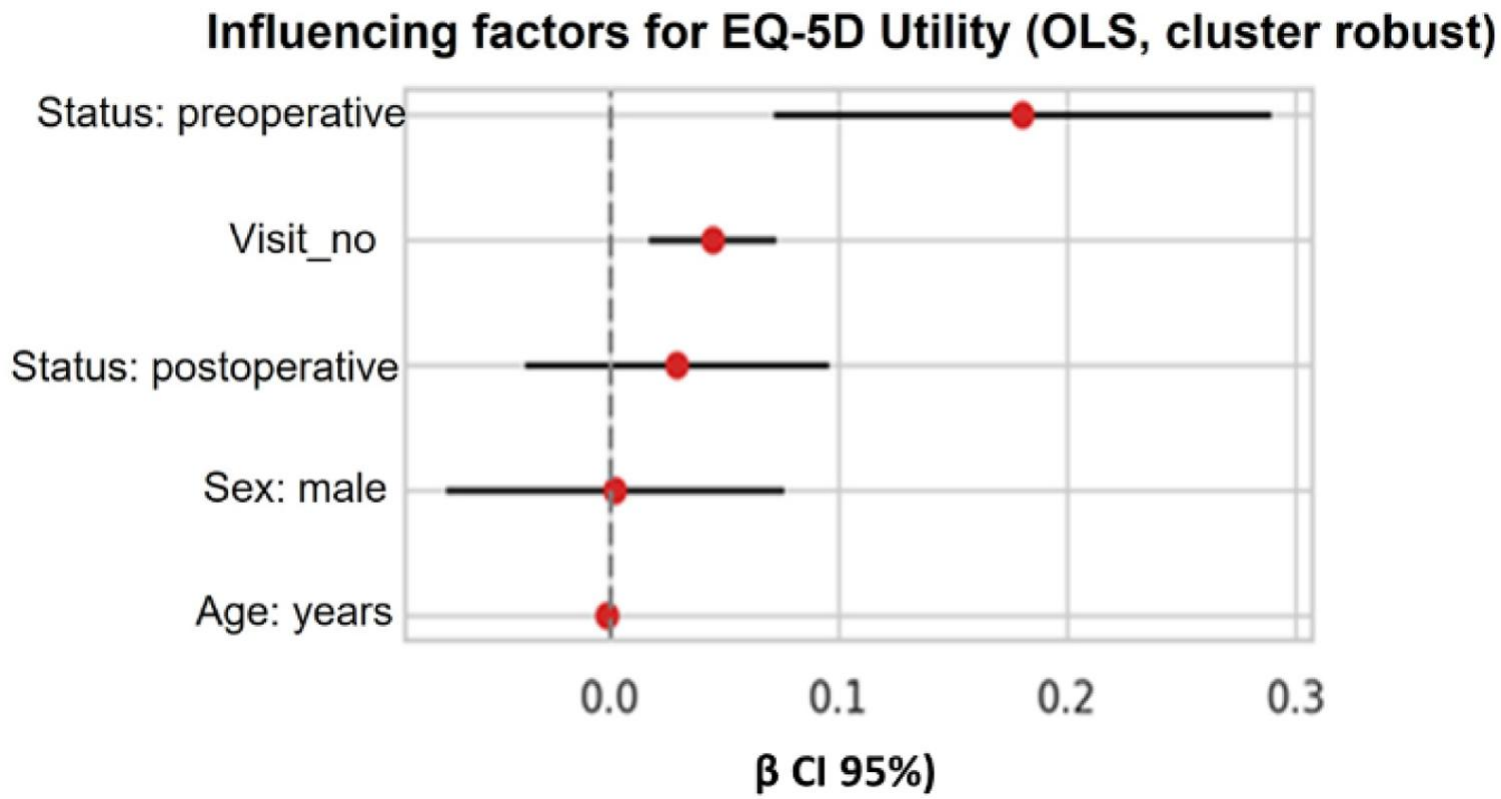
Forest plot of mixed-effects model *β*-coefficients (95% CI) for EQ-5D-5L index. Positive *β* indicates better HRQoL. Time since surgery shows positive effect; preoperative status (vs. postoperative reference) and female sex (vs. male reference) show negative associations.

Linear mixed-effects models revealed significant post-surgical HRQoL improvement across all outcomes (Figures 9a–c). Time since surgery increased EQ-5D-5L scores by *β* = 0.0005 per day (95% CI 0.0002–0.0008, *p* < 0.001), while preoperative status reduced scores by *β* = −0.15 (95% CI −0.25 to −0.05, *p* < 0.01). Female sex showed nonsignificant reduction (*β* = −0.08, 95% CI −0.18 to 0.02, *p* = 0.12) (Figures 9a–c). Similar patterns emerged for EQ-VAS and PHQ-4, with forest plots summarizing all coefficients (Figures 10–12).

#### Mixed-effects models

For in-depth analysis, linear mixed-effects models were used.

HRQoL (EQ-5D-5L and VAS) improved significantly with increasing time after surgery. Preoperative status (not female sex) predicted worse outcomes across models. Spearman correlations (*ρ*) reported due to non-normality (Shapiro–Wilk *p* < 0.001).

Time since surgery predicted HRQoL improvement (EQ-5D *β* = 0.0005/day, 95% CI 0.0002–0.0008), preoperative status reduced scores (*β* = −0.15, 95% CI −0.25 to −0.05), and female sex showed nonsignificant negative trends across outcomes. Forest plots detail all coefficients (Figures 10–12). Exploratory k-means clustering (*k* = 3) yielded three overlapping patterns (Pattern A, Pattern B, Pattern C). For description, we report their average trajectories; given overlap and sample size, no inferential interpretation is possible.

#### Clarification of EQ-VAS coding

All analyses were performed using the standard EQ-VAS 0–100 scale.

#### Predictors of psychological distress (PHQ-4 ≥6)

Using postoperative status as reference, preoperative patients had approximately double the odds of PHQ-4 ≥6 (OR≈2.0), corresponding to OR≈0.5 for postoperative vs. preoperative status. Male sex showed protective trend vs. female reference (OR <1), while age (OR≈1.0) and visit number (OR≈0.9) showed minimal effects. Cluster-robust standard errors accounted for within-patient correlation (Figure 11).

For the number of presentations, the odds ratio is approximately 0.9, indicating a slight, non-significant decrease in risk with an increasing number of presentations. The preoperative status shows an odds ratio of approximately 0.5, indicating a significantly reduced risk for PHQ-4 ≥6 in postoperative patients compared to preoperative ones. The effect is statistically significant, as the 95% CI does not overlap with 1.

Overall, the model allows for a nuanced examination of the determinants of health-related quality of life and can contribute to a more nuanced characterization of variables showed with HRQoL over time.

#### Cluster analysis

We performed principal components analysis (PCA) on z-standardized longitudinal scores from EQ-5D-5L index, EQ-VAS, and PHQ-4 total, including only patients with ≥2 visits (*n* = 67). The first two components explained 55% of variance and were subjected to k-means clustering (*k* = 3; mean silhouette width = 0.52), yielding three partially overlapping patterns:

- Pattern A: low baseline distress with improving HRQoL
- Pattern B: intermediate values with stable trajectories
- Pattern C: high initial HRQoL with subsequent deterioration and rising distress

These patterns are descriptive only and require validation in independent cohorts.

The following discussion interprets these findings in the context of existing cardiac surgery literature and their implications for psychosocial care.

#### Sex differences in odds estimates

Male patients exhibited lower odds of PHQ-4 ≥6 compared with female patients; however, this association was not statistically significant. Odds ratios quantify odds, not risk, and no conversion to risk ratios was performed.

#### Quality-of-life modelling

EQ-5D-5L index values were analysed using linear mixed-effects models. Model assumptions were checked visually, and variance components indicated appropriate fit for longitudinal data.

## Discussion

This longitudinal study provides a detailed characterization of HRQoL and psychological distress trajectories in patients with thoracic aortic disease. By adhering to a predefined analysis plan and systematically validating model assumptions, we offer a comprehensive observational description of temporal patterns and their associations with demographic and clinical variables. This approach enhances interpretability and methodological transparency, ensuring that reported associations reflect the structure of the real-world cohort rather than *post-hoc* analytical decisions. The primary findings include significant postoperative improvement in HRQoL and modest reductions in psychological distress, findings that align with previous studies in other surgical populations (8, 16). Previous studies have also demonstrated strong associations between health-related quality of life and clinical as well as behavioral factors in patients with aortic disease (20). Comparable reductions in anxiety and depressive symptoms after aortic surgical interventions have been reported previously, supporting the observed trajectory patterns in our cohort (21). These findings are consistent with recent longitudinal EQ-5D-based analyses in aortic disease populations. Sharples et al. (2024) reported heterogeneous but overall improving HRQoL trajectories in patients with thoracic aortic aneurysms, highlighting substantial inter-individual variability over time (18). Similarly, Torbjörnsson et al. (2025) demonstrated persistent but gradually improving quality of life and psychological outcomes following acute aortic dissection (4).

In comparison, our real-world outpatient cohort extends these findings by capturing both pre- and postoperative phases within routine clinical care, thereby illustrating not only recovery trajectories but also the elevated psychosocial burden in patients awaiting surgery. The observed heterogeneity across all three studies underscores the importance of repeated patient-reported outcome assessment rather than relying on single timepoint evaluations.

Preoperative status was consistently associated with lower HRQoL and higher distress across all models, with patients awaiting surgery demonstrating substantially greater psychological burden—including twice the odds of clinically relevant distress—compared with those in follow-up. This observation is consistent with established evidence showing that anticipation of surgical procedures is associated with increased psychological stress and anxiety, highlighting the importance of psychological preparation (22). Female sex showed trends toward worse HRQoL and higher distress, consistent with earlier research on sex-specific psychosocial responses (11, 12), although these associations did not reach statistical significance in this sample. Our descriptive baseline comparisons (Table 2) further indicate that patients selected for surgery differ from conservatively managed patients, particularly in HRQoL and pain levels, while psychological distress levels were comparable. These differences highlight selection mechanisms inherent to real-world surgical decision-making and reinforce the need for caution when interpreting longitudinal mixed-cohort analyses as if they were homogeneous comparative studies. The observed baseline disparities underscore that postoperative improvement reflects both recovery and preexisting clinical and psychosocial differences.

It is therefore essential to interpret this study not as a definitive comparison of homogeneous treatment groups but as an observational examination of routine care trajectories within a mixed pre- and postoperative TAD cohort. While the present analysis combines cross-sectional and longitudinal elements, a subset of patients contributed repeated measurements over time, allowing descriptive insight into within-patient trajectories. However, due to the real-world structure of follow-up and incomplete pairing of pre- and postoperative observations within individuals, the study was not designed to provide a fully balanced longitudinal pre-post comparison.

Instead, the applied mixed-effects modeling approach incorporates both between- and within-patient variation and represents a pragmatic strategy to analyze heterogeneous clinical follow-up data. This approach allows us to capture overall temporal trends while acknowledging that individual pre–post transitions cannot be interpreted in a strictly paired manner. The real-world composition of the sample—including patients who were never operated and those who underwent emergency procedures—accurately reflects outpatient clinical practice. Accordingly, the findings should be understood as descriptive and hypothesis-generating, illustrating clinically relevant psychosocial patterns rather than providing direct prescriptive guidance for individual treatment decisions.

The mixed-effects modeling approach allowed inclusion of all available data despite variable follow-up lengths and heterogeneous visit counts, with random intercepts accommodating within-patient correlation. Rigorous diagnostic checks—evaluation of linearity through residual plots, assessment of heteroscedasticity, and confirmation of low multicollinearity (VIF <2)—supported the validity of the modeling strategy. The use of *β*-coefficients for continuous outcomes and odds ratios for the binary PHQ-4 outcome ensured clear interpretability and avoided confusion between effect metrics.

Correlational analyses confirmed expected associations, with strong convergence between the EQ-5D-5L index and EQ-VAS (*ρ* = 0.75) and modest inverse correlations with PHQ-4. Exploratory PCA followed by k-means clustering identified three partially overlapping psychosocial patterns, including a subgroup with initially high HRQoL followed by deterioration and increasing distress. Such clustering approaches are increasingly recognized as valuable tools for identifying clinically meaningful subgroups and enabling more personalized patient care strategies (23). Although exploratory, these findings suggest heterogeneity in recovery patterns that may be underrecognized in mean-level analyses. The use of dimensionality reduction (explaining 55% of variance) and silhouette-based cluster selection (0.52) further strengthens transparency compared with prior exploratory approaches.

Several methodological strengths support the robustness of these observations. Validated screening tools (EQ-5D-5L and PHQ-4) ensured measurement reliability in a busy outpatient environment. The PHQ-4 has been specifically validated in surgical populations as a brief and feasible screening tool for perioperative psychological distress (24). The use of standardized patient-reported outcome measures in cardiac surgery research is well established, providing clinically meaningful insights beyond traditional clinical endpoints (25). While the PHQ-4 does not allow a detailed psychodiagnostic differentiation of anxiety and depressive disorders, it represents a validated ultra-brief screening instrument that is particularly suitable for high-throughput clinical settings. Thus, its use reflects a deliberate design choice balancing feasibility and systematic psychosocial assessment in routine cardiothoracic outpatient care rather than a methodological shortcoming. High data completeness—particularly the absence of missing PHQ-4 and EQ-VAS scores—reduced the likelihood of bias under the MAR assumption. The pre-specified analysis plan prevented data-driven model refinement, and sensitivity analyses confirmed the stability of results against outliers and alternative model specifications.

Nevertheless, several limitations must be considered. The retrospective single-center design limits generalizability beyond similar high-volume aortic centers. Although MAR assumptions were supported, residual confounding and differential follow-up related to psychosocial status cannot be excluded. Factors such as social vulnerability and socioeconomic status, which have been shown to influence outcomes after aortic surgery, were not available in this dataset and may represent additional sources of residual confounding (26). The dichotomized PHQ-4 threshold provides clinical interpretability but sacrifices granularity compared with continuous scores. Adjustment was limited to core demographic and clinical variables. However, none of the included patients had documented preexisting psychiatric diagnoses such as depression or anxiety disorders at the time of presentation. Likewise, no patient had received prior psychotherapy, psychopharmacological treatment (e.g., antidepressants or anxiolytics), or structured psychological support during the observation period. While this reduces the likelihood of major confounding by pre-existing mental health conditions, unmeasured psychosocial factors cannot be fully excluded.

This single-center observational study in a mixed pre- and postoperative cohort was not designed as a definitive comparative trial. Consequently, all subgroup contrasts are descriptive and hypothesis-generating. While such groupings might appear clinically intuitive, our sample size and the real-world nature of the cohort would have rendered fully adjusted subgroup comparisons statistically underpowered and potentially misleading. We therefore chose to model surgical status and time since surgery as covariates in mixed-effects models and to restrict subgroup analyses (e.g., operated vs. non-operated, dissection vs. aneurysm) to descriptive summaries. Similarly, the subgroup analysis of patients with aortic dissection vs. aneurysmal disease (Table 3) confirms that dissected patients constitute a clinically distinct group, with higher baseline HRQoL and lower pain levels despite broadly comparable demographic profiles. This is in line with previous findings demonstrating that patients with aortic dissection represent a distinct subgroup with specific quality-of-life profiles and recovery trajectories (27). However, because patients with acute dissection were predominantly captured after emergency repair, our data do not permit robust inferential pre- vs. postoperative comparisons within this subgroup, and these contrasts should be interpreted as descriptive only.

In summary, this observational analysis with explicit diagnostic checks demonstrates that patients with thoracic aortic disease generally experience postoperative improvements in HRQoL and reductions in psychological distress, although a substantial minority shows persistently elevated distress. These findings provide an important real-world perspective supporting the integration of structured psychosocial screening into routine aortic care pathways, particularly to identify vulnerable patients during preoperative phases or along heterogeneous recovery trajectories. Preoperative patients represent a key risk group warranting psychosocial assessment, and sex-related trends deserve further investigation. Routine use of brief PRO measures (EQ-5D-5L, PHQ-4) appears feasible and may support earlier detection of at-risk individuals. Prospective, multicenter studies with broader psychosocial data collection will be essential to validate and expand these findings.

### Clinical implications

Prior studies have reported reductions in anxiety and depressive symptoms following aortic surgery, and our findings align with these established postoperative trajectories (6, 28). Despite differences in study design and cohort composition, the overall pattern of psychological recovery after complex aortic procedures appears consistent across settings, and our real-world outpatient data extend this evidence to a mixed TAD population under routine follow-up conditions. These results highlight the value of integrating patient-reported outcomes into standard thoracic aortic care. Current clinical guidelines emphasize comprehensive longitudinal management of aortic disease, although structured psychosocial assessment is not yet routinely implemented in clinical practice (29). The observed associations between baseline demographic characteristics and longitudinal HRQoL and distress suggest that certain subgroups may carry a disproportionate psychosocial burden throughout the disease course. Even though these associations do not establish causality, they provide clinically relevant signals that may inform targeted surveillance or supportive care strategies in practice. Routine screening for HRQoL impairment and psychological distress may help clinicians identify patients with elevated burden during follow-up. Continuous monitoring of psychosocial factors throughout the perioperative course has been proposed as a key element of patient-centered cardiac care (30). These assessments should be viewed as complementary tools rather than predictive instruments for individual risk. By incorporating brief and feasible screening measures into clinical workflows, providers can better identify patients who may benefit from psychosocial support or closer monitoring, thereby enhancing overall patient-centered care. Integration of such assessments may also support rehabilitation strategies and improve long-term quality-of-life outcomes, as demonstrated in broader cardiac populations (31).

### Research implications

From a research perspective, the descriptive patterns emerging from the multivariable analyses and the exploratory PCA-based clustering provide a foundation for hypothesis generation. Future studies should prioritize prospectively defined analytical strategies, confirm the observed associations in independent cohorts, and integrate psychosocial variables more systematically to capture the complexity of patient-reported outcomes in thoracic aortic disease. Further research is needed to clarify the mechanisms linking demographic and clinical characteristics with HRQoL and psychological distress and to determine whether targeted interventions can mitigate deterioration in these outcomes over time. Prospective study designs with predefined endpoints and methodologically robust statistical frameworks will be essential to translate these exploratory observations into clinically actionable evidence. Structured psychosocial evaluation should also be incorporated into future research settings. Preoperative screening may help identify vulnerable periods requiring closer attention, and longitudinal monitoring can reveal heterogeneous recovery trajectories that may benefit from individualized follow-up strategies. Multicenter prospective studies will be needed to determine whether targeted psychosocial interventions can meaningfully modify the observed patterns and improve patient-centered outcomes.

## Limitations

This study has several limitations that should be considered when interpreting the findings. First, the observational design precludes causal inference, and residual confounding cannot be entirely excluded despite adjustment for core demographic and clinical variables. Second, the study may be underpowered to detect smaller effect sizes, particularly regarding non-significant trends such as sex-specific differences or potential interactions. Third, the exploratory clustering analysis was restricted to patients with at least two visits, and these results should therefore be interpreted with caution and require validation in independent cohorts.

Fourth, although the use of the PHQ-4 and EQ-5D-5L enabled efficient and repeated assessment in a routine outpatient setting, these brief instruments provide limited psychodiagnostic depth and may not capture more subtle or disorder-specific psychological changes. Fifth, while no patient in the cohort had documented pre-existing psychiatric diagnoses or received psychopharmacological or psychotherapeutic treatment during the observation period, unmeasured psychosocial or clinical factors may still have influenced the results.

Finally, the single-center design limits generalizability to other healthcare systems and patient populations. The findings should therefore be interpreted as hypothesis-generating and require confirmation in larger, prospective, multicenter studies.

## Conclusion

In this longitudinal real-world cohort of patients with thoracic aortic disease, preoperative individuals exhibited lower HRQoL and higher psychological distress compared with postoperative patients. Operated patients demonstrated higher baseline HRQoL than non-operated patients, while those presenting with dissection showed better HRQoL but higher pain levels than patients with aneurysmal disease. Given the single-center observational design, the mixed structure of pre- and postoperative visits, and the limited sample size, these subgroup contrasts—as well as the heterogeneous trajectories observed, including a subgroup with persistent or worsening distress—must be viewed as exploratory and hypothesis-generating rather than definitive. Future prospective studies in larger and more diverse cohorts are needed to validate these patterns and clarify their implications. Such work should also establish whether structured, routine integration of brief PRO instruments (EQ-5D-5L, PHQ-4) into aortic care pathways can reliably identify psychosocially vulnerable patients and support early, targeted interventions.

## Data Availability

The original contributions presented in the study are included in the article/Supplementary Material, further inquiries can be directed to the corresponding author/s.

## References

[1] Sato M Mutai H Yamamoto S Tsukakoshi D Furuhashi K Ichimura H. Characteristics of longitudinal changes in quality of life and associated factors in patients post cardiac and thoracic aortic surgery. J Patient Rep Outcomes. (2024) 8:111. 10.1186/s41687-024-00787-9 39325084 PMC11427642

[2] Eichenberg C Hübner L Fiegl J Weihs V Huber K. Psychokardiologie: Das Herz als projektionsort psychischer konflikte. Dtsch. Ärztebl. (2019) 18(8):370–3.

[3] Liberzon I Abelson JL Amdur R King AP Cardneau JD Henke P. Increased psychiatric morbidity after abdominal aortic surgery: risk factors for stress-related disorders. J Vasc Surg. (2006) 43(5):929–34. 10.1016/j.jvs.2006.01.026 16678685

[4] Torbjörnsson E Nilsson O Stenman M Olsson C Steuer J Hultgren R. Quality of life, anxiety and depression after acute type B aortic dissection. Ann Vasc Surg. (2025) 112:157–65. 10.1016/j.avsg.2024.11.097 39694189

[5] Ilonzo N Taubenfeld E Yousif MD Henoud C Howitt J Wohlauer M. The mental health impact of aortic dissection. Semin Vasc Surg. (2022) 35(1):88–99. 10.1053/j.semvascsurg.2022.02.005 35501046

[6] Vanmaele A Branidis P Karamanidou M Bouwens E Hoeks SE de Bruin JL. Evolution of quality of life, anxiety, and depression over time in patients with an abdominal aortic aneurysm approaching the surgical threshold. BJS Open. (2024) 9(1):zrae150. 10.1093/bjsopen/zrae150 39792053 PMC11720167

[7] Lyttkens L Wanhainen A Svensjö S Hultgren R Björck M Jangland E. Systematic review and meta-analysis of health-related quality of life and reported experiences in patients with abdominal aortic aneurysm under ultrasound surveillance. Eur J Vasc Endovasc Surg. (2020) 59(3):420–7. 10.1016/j.ejvs.2019.07.021 31928908

[8] Kroenke K Spitzer RL Williams JBW Löwe B. An ultra-brief screening scale for anxiety and depression: the PHQ-4. Psychosomatics. (2009) 50(6):613–21. 10.1176/appi.psy.50.6.613 19996233

[9] Kerper LF Spies CD Tillinger J Wegscheider K Salz AL Weiss-Gerlach E. Screening for depression, anxiety, and general psychological distress in pre-operative surgical patients: a psychometric analysis of the PHQ-4. Clin Health Promot. (2014) 4:5–14. 10.29102/clinhp.14002

[10] Adzrago D Walker TJ Williams F. Reliability and validity of the patient health questionnaire-4 scale and its subscales of depression and anxiety among US adults. BMC Psychiatry. (2024) 24:213. 10.1186/s12888-024-05665-8 38500115 PMC10949792

[11] Thijssen CGE Vahl AC Verhagen HJM Dekker S Bons LR Gökalp AL. Health-related quality of life and lived experiences in males and females with thoracic aortic disease and their partners. Open Heart. (2020) 7(2):e001419. 10.1136/openhrt-2020-001419 33033116 PMC7545641

[12] Olsson C. Health-Related quality of life in thoracic aortic disease. Aorta. (2013) 1(2):116–22. 10.12945/j.aorta.2013.13-028 PMC4682737 26798688

[13] Korbmacher B Ulbrich S Dalyanoglu H Lichtenberg A Schipke JD Franz M. Perioperative and long-term development of anxiety and depression in CABG patients. Thorac Cardiovasc Surg. (2013) 61(08):676–81. 10.1055/s-0032-1328464 23344765

[14] Botzet K Dalyanoglu H Schäfer R Lichtenberg A Schipke J Korbmacher B. Anxiety and depression in patients undergoing mitral valve surgery: a prospective clinical study. Thorac Cardiovasc Surg. (2018) 66(07):530–6. 10.1055/s-0037-1604461 28780764

[15] Erbel R Aboyans V Boileau C Eggebrecht H Evangelista A Falk V. 2014 ESC guidelines on the diagnosis and treatment of aortic diseases. Eur Heart J. (2014) 35(41):2873–926. 10.1093/eurheartj/ehu281 25173340

[16] Kappetein AP Gittenberger-de Groot AC. Quality of life after aortic surgery. J Thorac Cardiovasc Surg. (1997) 113(1):194–203. 10.1016/S0022-5223(97)70395-39011690

[17] von Känel R Hari R Schmid JP Saner H. Psychosocial resources and physical recovery in patients undergoing cardiac surgery. Heart. (2021) 107(13):1069–74. 10.1136/heartjnl-2020-31867533109710

[18] Sharples LD Anagnostopoulou V Pouncey AL Freeman C McCarthy A Gray J. Large on behalf of the ETTAA study investigators. Longitudinal health-related quality of life in people with thoracic aortic aneurysms. Br J Surg. (2024) 111(9):znae228. 10.1093/bjs/znae228 39258491 PMC11387963

[19] Herdman M Gudex C Lloyd A Janssen M Kind P Parkin D. Development and preliminary testing of the new five-level version of EQ-5D (EQ-5D-5L). Qual Life Res. (2011) 20(10):1727–36. 10.1007/s11136-011-9903-x 21479777 PMC3220807

[20] Tu J Wang F Yin F Zhang L Zhao B Zhou J. The relationship between quality of life and health promotion behavior in patients with type B aortic dissection: a cross-sectional study. J Cardiothorac Surg. (2023) 18:23. 10.1186/s13019-023-02124-5 36639794 PMC9838059

[21] Mendes CA Wolosker N Fioranelli A Mello RAF Portugal MFC Silva MFAD. Anxiety and depression scores in patients subjected to aortic and iliac aneurysm repair procedures. Rev Assoc Med Bras. (2021) 67(5):747–52. 10.1590/1806-9282.20210187 34550267

[22] Cochran TM. Psychological preparation of patients for surgical procedures. Patient Educ Couns. (1984) 5(4):153–8. 10.1016/0738-3991(84)90174-5 10317451

[23] Dou B Moons P. Cluster analysis in patients with heart diseases: the cornerstone for personalized and tailored care. Eur J Cardiovasc Nurs. (2025) 24(36):911–2. 10.1093/eurjcn/zvaf070 40323337

[24] Tillinger J. Der PHQ-4 zur messung von depressivität und angst bei operativen patienten der anästhesieambulanz (Dissertation). Charité – Universitätsmedizin Berlin (2015).

[25] Tully PJ. Quality-of-life measures for cardiac surgery practice and research: a review and primer. J Extra Corpor Technol. (2013) 45(1):8–15. 10.1051/ject/201345008 23691778 PMC4557469

[26] Harik L Leith J Rahouma M Cancelli G Rossi CS Soletti G. Association between social vulnerability and clinical outcomes after proximal aortic surgery. Eur J Cardiothorac Surg. (2025) 67(7):ezaf217. 10.1093/ejcts/ezaf217 40591461 PMC12288956

[27] Lin XF Xie LF Zhang ZF He J Xie YL Dai XF. Quality of life in young patients with acute type A aortic dissection in China: comparison with Marfan syndrome and non-Marfan syndrome. BMC Cardiovasc Disord. (2024) 24(1):132. 10.1186/s12872-024-03740-2 38424531 PMC10905939

[28] Sharples LD Anagnostopoulou V Pouncey AL Freeman C McCarthy A Gray J. Longitudinal health-related quality of life in people with thoracic aortic aneurysms. Br J Surg. (2024) 111(9):znae228. 10.1093/bjs/znae228 39258491 PMC11387963

[29] Isselbacher EM Preventza O Hamilton Black J Augoustides JG Beck AW Bolen MA. 2022 ACC/AHA guideline for the diagnosis and management of aortic disease: a report of the American Heart Association/American College of Cardiology joint committee on clinical practice guidelines. CirculationVolume. (2022) 146(24):e334–482. 10.1161/CIR.0000000000001106 PMC9876736 36322642

[30] Callus E Pagliuca S Bertoldo EG Fiolo V Jackson AC Boveri S. The monitoring of psychosocial factors during hospitalization before and after cardiac surgery until discharge from cardiac rehabilitation: a research protocol. Front Psychol. (2020) 11:2202. 10.3389/fpsyg.2020.02202 33117210 PMC7550819

[31] Delgado-Calderon M Jimenez-Ortega ES Delgado-Calderón M Jiménez-Ortega LE Ladisa M Camacho-Vega JC. Cardiac rehabilitation for workers with ischemic heart disease: benefits for cardiovascular health and quality of life. Medicine. (2025) 104(36):e44019. 10.1097/MD.0000000000044019 40922291 PMC12419251

