# Supplementary material for: Anxiety, depression and health-related quality of life in patients with thoracic aortic disease: a longitudinal study in a cardiothoracic outpatient clinic
Source: Front Cardiovasc Med. 2026 Jun 29;13:1715132. doi: 10.3389/fcvm.2026.1715132 (PMC13357163; doi:10.3389/fcvm.2026.1715132)
Supplement: Supplementary file 1 [file Datasheet1.pdf]

Supplementary Appendix: Detailed Statistical Methods

Distributional Assumptions
Normality of outcome variables (EQ-5D-5L index, EQ-VAS, PHQ-4) was assessed using Shapiro–Wilk tests and visual inspection of histograms and Q-Q plots. All primary outcomes deviated significantly from normality ($p < 0.001$), justifying the use of non-parametric and robust modeling approaches.

Correlation Analysis
Bivariate associations were evaluated using Spearman's rank correlation coefficient (ρ), given non-normal distributions and ordinal properties of some variables.

Mixed-Effects Models
Continuous outcomes were analyzed using linear mixed-effects models (LMMs) with random intercepts for patient ID to account for within-patient correlation due to repeated measurements.

Model structure:
For continuous outcomes (EQ-5D-5L index, EQ-VAS, PHQ-4 total score), linear mixed-effects models were used.
Each model included the following fixed effects:

- time since surgery (continuous variable),
- biological sex,
- surgical status (preoperative vs. postoperative),

as well as a patient-specific random intercept to account for repeated measurements within individuals.
This means that each patient has an individual baseline level, while the model estimates overall effects of time, sex, and surgical status across the cohort.
For transparency, the model can also be expressed in statistical notation as:
Outcome ~ time_since_surgery + sex + surgical_status + (1 | patient_ID)

Logistic Regression
The binary outcome (PHQ-4 $\geq$ 6) was analyzed using logistic regression models with cluster-robust standard errors to account for repeated observations within individuals.

Model Specification
Model selection followed a predefined analysis plan. Only main effects were included to avoid overfitting given the observational design and sample size.

Multicollinearity and Diagnostics
Variance inflation factors (VIF) were calculated for all predictors, with values < 2 indicating no relevant multicollinearity. Residual diagnostics were assessed visually to confirm model adequacy.

Sensitivity Analyses
Sensitivity analyses excluding statistical outliers were performed and showed no relevant deviation from the primary results.

All analyses were conducted using R (version 4.3; packages: lme4, stats, cluster).
